# How to Make Both Ends Meet

**Published:** 1893-06-03

**Authors:** 


### How to Make Both Ends Meet.

(An Interview with the Rev. Canon Palmer.)

We have long been of opinion that the Hospital Sunday collections ought to raise £100,000 a year. But we have also felt that the clergy and their congregations have not altogether shared our enthusiasm. Why this apparent lack of zeal, we asked ourselves? With the view of answering the question correctly we felt it best to consult some of the clergy themselves. The Rev. Canon Palmer, Rector of Newington, very kindly and genially consented to be cross-examined on behalf of himself and his brethren; and we now give our clerical readers and their congregations the benefit of his sometimes witty and always wise advice.

The great problem, says Canon Palmer, is "how to make both ends meet." On this point we are all quite prepared to agree with him. But how is the problem to be solved? The rev. Canon does not, like Socialists and other crude speculators, go promptly and directly for the rich, and insist that they shall make up the deficit out of their vast superfluous resources. On the contrary, he has a very tender feeling towards the rich, and that not because he is the rector of a rich parish, but because he is a man of genial good sense, and has a reasonable knowledge of the world.

The Carcase and the Vultures.

The rich, thinks Canon Palmer, are very much like the "carcase" mentioned in the Scriptures, and the hungry eagles, representing all sorts of charities, gather themselves together to the feast with a right good will. But, says this reverend sympathiser, "the carcase is not passive." It sometimes shows an uncharitable desire to lay hold of a stick and drive the greedy vultures away. Canon Palmer, with a merry twinkle, admits that he experiences a certain amount of fellow feeling for the carcase and the stick. He expresses the opinion that the rich ought not to be "spendthrifts" even in charity; for he does not doubt that "charitable" beggars would ruin a rich man quite as willingly as beggars of any other class.

But notwithstanding all his sympathy with the rich and their worries, Canon Palmer thinks that they might with advantage give a little more to hospitals than they do; and that because hospitals are so pre-eminently necessary, and do such noble, and altogether indispensable work. We venture to commend to the rich these very considerate and very sympathetic suggestions.

The Wealthy Poo

Canon Palmer gave expression to one idea which we admit had not struck us so forcibly before. He was talking about the poor, and how very much more they might do for hospitals if they would; and this is the remark which struck us as being so worthy of consideration: "The aggregate wealth of the labouring classes is enormous, as statistics prove; and the aggregate of their contributions to hospitals ought to be enormous too." This strikes us as being worthy of the serious consideration of all those who charge themselves with the responsibility of supporting hospitals. Here is an undoubted fact, that the aggregate wealth of the labouring classes is enormous. A little taken from an enormous mass makes small difference to the mass; but it might and would make all the difference in the world to such institutions as the hospitals.

Good Fellows who give Pence not Pounds.

The Rector of Newington speaks with special authority on this point because he is rector, not of a rich, but of one of the largest and poorest parishes in London. He avows himself quite convinced that the working people would give, and give freely, if the clergy and other supporters of hospitals would only have the intelligence to fit the right sort of "tap" into the wine cask of their enormous resources. "The poor give in pennies, not in pounds," says the Canon. Will the hospitals send out gleaners to gather up the pennies, or will they insist—as before—upon living on the sheaves and argosies of the rich?

The toilers, according to their reverend apologist, are thoroughly good fellows at heart, and will give if only they are allowed to give in their own way. They must, however be allowed to give in their own way, says the Canon; and he has a practical scheme all cut and ready, by means of which they can give in their own way. He proposes to make the Church and her workers the unpaid collectors of the toilers' hospital pence. An excellent scheme it is, in our judgment, and though it is too late to be put into operation before the Hospital Sunday immediately ensuing, the prompt and energetic Canon proposes to make an experiment with it in his own parish on behalf of the Sunday Fund collection of 1894.

A New Scheme.

This is Canon Palmer's proposal in briefest outline. The mission women and district visitors of the church now collect money from the working classes for various provident funds. He feels quite sure that they would be very enthusiastically willing to collect for a hospital fund, and that the poor would be equally enthusiastic in giving their periodical pence to such a fund. Some two or three months before next year's collection, the Canon proposes to distribute leaflets among all the poor of his parish, and a few weeks immediately preceding the Sunday itself he will provide the mission women and district visitors with collecting cards, and they will go round weekly for the money. Whatever sum is collected will be added as a separate and distinct amount to the collections taken in church on the Sunday. This scheme seems to us to be simplicity itself, and, so far as we can see, there is no reason why it should not be applied in every parish and congregation within the metropolitan area in the very next year—1894.

Two Advantages.

There are two incidental advantages which Canon Palmer thinks would result from the adoption of this scheme. The poor would, in the first place, when asked to pay, make known their grievances against hospitals just as the British Commons in the olden times used to demand from the King a redress of grievances before they would consent to be taxed. We are not, thinks Canon Palmer, to commit the serious mistake of supposing that no grievances exist. He insists that a good many grievances do exist, and that not a few of them are well founded. Now we are quite at one with this contention. Nobody knows better than we do how well hospitals are managed in the main, and how excellently their work is done. But it is to be remembered that a very great deal of it is done by young and inexperienced men and women. Now inexperienced men and women are impulsive and foolish sometimes in spite of all that can be done and said to prevent them. But we are anxious to reduce their folly and impulsiveness to a minimum, and if the hospitals learn to depend upon the poor for support, they will also have to learn to compel their officials to treat the poor with respect.

But there is another and much more pleasing aspect of this proposed collection of hospital pence. Whilst one here and there has a grievance and longs to tell it, there are scores and hundreds who are brimming over with gratitude for the benefits they have received at hospitals, and do not know how to say enough in their praise. These latter will communicate to the district visitors their own enthusiasm, and the visitors will spread it through the congregation. It seems certain that if Canon Palmer's suggestions were thoroughly and intelligently carried out, not only would the Sunday Fund collection be increased by the pence of the poor, but the better classes themselves would be so much better instructed and so much more enthusiastic, that they could hardly fail to largely increase their generous contributions. We must confess to a considerable amount of admiration for Canon Palmer's simple and comprehensive scheme; and we hope that many other clergymen in the metropolis will give the subject their careful consideration, and communicate with us personally or by letter before the end of the present year.

